# Is Prescription Nonredemption a Source of Poor Health Among the Roma? Cross-Sectional Analysis of Drug Consumption Data From the National Health Insurance Fund of Hungary

**DOI:** 10.3389/fphar.2021.616092

**Published:** 2021-03-09

**Authors:** Bayu Begashaw Bekele, Nouh Harsha, László Kőrösi, Ferenc Vincze, Árpád Czifra, Róza Ádány, János Sándor

**Affiliations:** ^1^ Doctoral School of Health Sciences, University of Debrecen, Debrecen, Hungary; ^2^ Department of Public Health, College of Health Sciences, Mizan Tepi University, Mizan Aman, Ethiopia; ^3^ Department of Public Health and Epidemiology, Faculty of Medicine, University of Debrecen, Debrecen, Hungary; ^4^ Department of Financing, National Health Insurance Fund, Budapest, Hungary; ^5^ MTA-DE Public Health Research Group, University of Debrecen, Debrecen, Hungary

**Keywords:** prescription non-redemption, Roma people, cardiovascular drugs, alimentary tract drugs, anti-infective agents

## Abstract

**Background:** The health status of the Roma is inferior to that of the general population. The causes of poor health among this population are still ambiguous, but they include low utilization of healthcare services. Our study aimed to investigate prescription redemptions in segregated Roma colonies (SRC) where the most disadvantaged quartile of Roma people are living.

**Methods:** A cross-sectional study was carried out with data obtained from the National Institute of Health Insurance Fund Management in the settlements belonging to the study area of the “Public Health-Focused Model Program for Organizing Primary Care Services.” The study included 4,943 residents of SRC and 62,074 residents of the complementary area (CA) of the settlements where SRC were located. Crude and age- and sex-standardized redemption ratios for SRC and CA were calculated for each Anatomic Therapeutic Chemical (ATC) group and for the total practice by ATC group. Standardized relative redemptions (RR) with 95% confidence intervals were calculated for SRC, with CA as a reference.

**Results:** The crude redemption ratios were 73.13% in the SRC and 71.15% in the CA. RRs were higher in the SRC than in the CA for cardiovascular, musculoskeletal system, and alimentary tract and metabolism drugs (11.5, 3.7, and 3.5%, respectively). In contrast, RRs were lower in the SRC than in the CA for anti-infective agents (22.9%) due to the poor redemption of medicines prescribed for children or young adults. Despite the overall modest differences in redemption ratios, some ATC groups showed remarkable differences. Those include cardiovascular, alimentary and musculoskeletal drugs.

**Conclusion:** Redemption of prescriptions was significantly higher among Roma people living in SRC than among those living in CA. The better redemption of cardiovascular and alimentary tract drugs was mainly responsible for this effect. These findings contradict the stereotype that the Roma do not use health services properly and that prescription non-redemption is responsible for their poor health.

## Introduction

Primary medication non-adherence or non-redemption of prescriptions occurs when patients do not dispense the new medications written by their health care providers (Adams and Stolpe, 2016). The World Health Organization (WHO) considers drug redemption to be an essential indicator of patient care and utilization of health care services among patients in primary health care (PHC) (WHO, 1993). Although better redemption of prescriptions is necessary for good health outcome, a recent meta-analysis revealed that the pooled primary medication nonadherence among patients with chronic diseases was 17% (Cheen et al., 2019). This escalates the negative outcomes of diseases and exposes patients to extra direct and indirect health expenditures (Jackson et al., 2014; Kleinsinger, 2018).

Ethnicity, gender, age, geographical location, socioeconomic status, and character of chronic disease have been shown to affect medication redemption (Butler et al., 2011; Fitzmaurice et al., 2019; Gast and Mathes, 2019). In addition, lack of motivation, low understanding of the severity of diseases, low education level, and poor cognitive ability reduce redemption ratios (Adams et al., 2015; Adams and Stolpe, 2016; Aznar-Lou et al., 2017). Medication- or drug-related barriers including side effects, complex combinations of drugs, inconvenient application, cost, and time requirement push patients to not fill prescriptions (Davidson et al., 2019). Other barriers include poor communication between the patient and practitioners, and asymptomatic diseases (Cooper et al., 1982; Gellad et al., 2009).

The Roma constitute the largest European ethnic minority, and they account for 1.7% of the population (European Commission, 2011). They originated from the northwestern Indian subcontinent and arrived in Europe between the 10th and the 12th century. Later, they were distributed throughout Europe (Gresham et al., 2001), but remained a marginalized social group with a disadvantaged health status (Ádány, 2014).

As has been convincingly demonstrated, the misuse of primary and secondary healthcare services is a risk factor for poor health among this population (Ekuklu et al., 2003; Colombini et al., 2012; Jakab et al., 2017; Sándor et al., 2018). Because of unresolved methodological problems of ethnicity-related health studies (identification of members of ethnic minorities) in the European legal environment (Janka et al., 2018), the determinants of health care use have not been explored in detail. Consequently, there are no effective interventions for this risk factor (Janka et al., 2018), and the perceived misuse contributes to the development of a blaming attitude toward the Roma among health professionals, which becomes a distinct obstacle for targeted interventions (Dougherty, 1993; Miranda et al., 2019). Unfortunately, primary medication adherence as a substantial indicator of appropriate healthcare use has not yet been investigated properly among the Roma.

According to a recent investigation, the Roma constitute approximately 9% of the total population in Hungary (Teller, 2010; Pásztor et al., 2016). Their most vulnerable stratum, representing 21.8% of the Hungarian Roma population, is living in segregated Roma colonies (SRC) (Teller, 2010). The substantial health inequality between the Roma and the general population has been demonstrated in many contexts in Hungary and other European countries (European Union Agency for Fundamental Rights, 2009a; Balkowski and Paweł Czyba, 2011; Ádány, 2013).

The legal opportunity to investigate the health of Roma people living in SRC has recently been established in Hungary by elaborating the transformation of investigations comparing Roma people to non-Roma people at the individual level to geographical inequality analysis, in which the group of Roma people living in SRC is compared to groups of people living in the same settlement’s complementary area (CA). This approach could describe the excess premature mortality among Roma people in SRC and the restricted availability of secondary care for this population (Sándor et al., 2018).

The National Institute of Health Insurance Fund Management (NHIFM) is an institution that covers the whole country. It is contracted with each general practitioner (GP) and with each pharmacy, and it centrally registers drug prescriptions and redemptions. Combining the drug consumption database of the NHIFM (Harsha et al., 2019a) and the legal opportunity for SRC investigation, an investigation of primary medication adherence among Roma people in SRC in Hungary is feasible without violating Roma rights.

The objectives of our study were to utilize this opportunity 1) by investigating prescription redemption among Roma people living in SRC and people living in the CA of the study area, 2) by assessing age- and sex-standardized relative redemption ratios for SRC, in order to, 3) support the formulation of health policy by exploring an important determinant of the population-level health status of the most vulnerable stratum of Roma people.

## Materials and Methods

### Setting

This study was a part of the “Public Health Focused Model Program for Organising Primary Care Services Backed by a Virtual Care Service Center” program in Hungary (Ádány, 2013). The main aim of this program was to reorganize preventive service delivery at PHCs by the GP team and to provide improved care without discriminating against the Roma people (Sándor et al., 2016). This program was conducted in 21 general medical practices from Borsod-Abauj-Zemplen, Heves, Jasz-Nagykun-Szolnok, and Hajdu-Bihar counties located in northeastern Hungary in the period of 2012–2017.

Applying the definition of SRC as a segregated part of the settlement with at least four disadvantaged residential units (apartment/house/block of rooms/hut), with a lower housing condition and poorer environmental quality than the CA (Kósa et al., 2009; Sándor et al., 2018), a survey was carried out to identify addresses belonging to the SRCs as a part of our presented study by epidemiologist of the intervention program. Because, GP knew addresses of each patient, they could classify their clients as SRC inhabitant or CA inhabitant. In the intervention area, there were 67,017 residents. Among these residents, the numbers of inhabitants living in SRC and in the CA were 4,943 and 62,074, respectively.

Our investigation was carried out in 2012 before the interventions had been launched.

### Data Sources

Data were provided by the NIHIFM. The unit of analysis was the prescription written by the health care provider and redeemed by the patient during the calendar year of 2012 before the interventions of the program had been launched (Sándor et al., 2016). The age- (applied age groups: 0–17, 18–24, 25–44, 45–64, and 65 years and above) and sex-specific nominators and denominators of the prescription redemptions were computed for the SRC and the CA. The prescribed medications were classified according to the WHO, 2012 version of the Anatomic Therapeutic Chemical (ATC) first level classification of drugs (WHO, 2011). Prescriptions written for all age groups were included in the analysis.

### Statistical Analysis

The crude redemption ratios were calculated by age, sex, and ATC group for the SRC, the CA, and Hungary. Indirect standardization was applied to ensure the comparability of SRC- and CA-specific indicators. Age- and sex-specific expected proportions of redeemed prescriptions were calculated by age- and sex-specific numbers of written prescriptions and the national reference redeemed-to-written ratio for all strata (Harsha et al., 2019a; Harsha et al., 2019b). To control for the confounding effect of the demographic factors, standardized redemption ratios (SRRs) were calculated by dividing accumulated age- and sex-specific numbers of observed redemptions by accumulated age- and sex-specific numbers of expected redemptions of prescribed drugs for both the SRC and the CA.

Relative redemption (RR), the ratio of SRRs for the SRC to SRRs for the CA, along with the corresponding 95% confidence interval (95% CI), was calculated for each ATC group and for the total practice. Attributable numbers of redemptions were calculated as the product of the difference in SRRs for the SRC and CA and the expected number of redemptions in the SRC.

## Results

**FIGURE 1:**
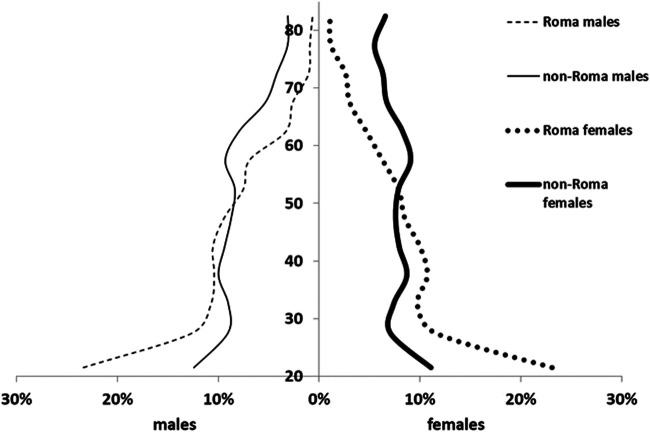
Demographic structures of investigated populations living in segregated Roma colonies (SRC) and complementary areas (CA).

Males constituted 50.23% of the SRC population and 48.28% of the CA population. In the SRC, 38.94% of the inhabitants were children aged 0–17 years, compared to 16.07% in the CA. The proportion of people aged 65 years and above was 3.7% in the SRC and 17.16% in the CA. The differences in age and sex distribution between the two populations were statistically significant by the chi-squared test (*p* < 0.001) (Figure 1).

In 2012, the national crude redemption ratio was 66.8% (84,323,051/126,223,796). Variations across age groups were noted, with higher redemption ratios reported for children and the elderly population (69.78 and 68.36%, respectively). Females redeemed medications more often than males. Differences observed by age and sex were statistically significant (Supplementary Table S1). By ATC group (Supplementary Table S2), the highest redemption ratios in the country were reported for anti-infective agents for systemic use (76.80%) and antiparasitic products, insecticides and repellents (75.33%), while the lowest ratios were reported for dermatological drugs (63.07%) and cardiovascular system drugs (63.12%) (details on demographic stratum-specific redemption ratios by ATC class are provided in Supplementary Tables S3A,V).

In the SRC, the crude redemption ratio for total practice was 73.13% (46,107 prescriptions were written, and 33,720 were redeemed) (Table 1). In the CA, the redemption ratio was 71.15% (900,901 prescriptions were written, and 640,950 prescriptions were redeemed). Redemption was below the average for children in both populations (58.08% in the SRC and 68.23% in the CA). Elderly adults aged 65 years and above reported better redemption in both the SRC and CA (74.69% and 72.36%, respectively). The SRC redemption ratios in each demographic group except for children and youngest adults were higher than those in the reference population. Redemption of cardiovascular drugs was relatively high in the SRC (74.92%) but was lowest in the CA (67.15%). In contrast, redemption of anti-infective agents for systemic use was relatively high in the CA (74.67%) but was lowest in the SRC (57.60%).

**TABLE 1:** Crude prescription redemption ratio by sociodemographic characteristic and ATC class for segregated Roma colonies and complementary area.

		Segregated Roma colonies			Complementary area			*p*-value^a^
		Written prescriptions	Dispensed prescriptions	Dispensed percentage	Written prescriptions	Dispensed prescriptions	Dispensed percentage	
Age category in years	0–17	8,839	5,134	58.08	47,172	32,187	68.23	<0.001
	18–24	1,284	671	52.23	14,130	9,122	64.56	<0.001
	25–44	7,884	5,933	75.26	80,208	57,453	71.63	0.006
	45–64	19,923	15,874	79.67	350,836	246,581	70.28	<0.001
	65 and above	8,177	6,108	74.69	408,555	295,607	72.36	0.062
Sex	Male	17,662	12,703	71.90	346,089	245,279	70.80	0.217
	Female	28,445	21,017	73.88	554,812	395,671	71.3	<0.001
A-alimentary tract and metabolism		6,581	5,107	77.60	132,702	99,469	74.96	0.070
B-blood and blood forming organs		2,493	1,894	75.97	66,839	49,669	74.31	0.476
C-cardiovascular system		13,356	10,156	74.92	409,505	274,969	67.15	<0.001
D-dermatologicals		1,029	685	66.57	12,384	8,575	69.24	0.443
G-genito-urinary system and sex hormones		201	159	79.10	5,846	4,557	77.95	0.892
H-systemic hormonal preparations^b^		406	305	75.12	9,651	7,482	77.53	0.684
J-anti-infectives for systemic use		6,451	3,716	57.60	53,999	40,324	74.67	<0.001
M-musculo-skeletal system		5,143	3,936	76.53	71,036	52,403	73.77	0.094
N-Nervous system		3,369	2,612	77.53	64,578	49,299	76.34	0.563
P-antiparasitic products, insecticides and repellents		32	20	62.50	539	416	77.18	0.470
R-respiratory system		5,125	3,819	74.52	62,137	45,222	72.78	0.289
S-sensory organs		505	316	62.57	6,012	4,297	71.47	0.074
V-various		1,216	995	81.83	5,673	4,268	75.23	0.076
Total		46,107	33,720	73.13	900,901	640,950	71.15	<0.001

^a^ By chi-square test.

^b^ Excludingex hormones and insulins.

The SRR for total practice was 1.027 [95% CI: 1.016–1.038] in the SRC and 0.999 [95% CI: 0.996–1.001] in the CA (Table 2). The RR was 1.028 [95% CI: 1.018–1.038]. Thus, a 2.8% better redemption was observed in the SRC than in the CA. Significant differences were reported for alimentary tract and metabolism-related drugs (RR = 1.035), cardiovascular agents (RR = 1.115), musculoskeletal system drugs (RR = 1.037) and drugs belonging to the various ATC group (RR = 1.088), which were better redeemed in the SRC. Conversely, anti-infectives for systemic use (RR = 0.771, 95% CI: 0.753–0.791) and drugs of sensory organs (RR = 0.875) showed significantly lower redemption in the SRC.

**TABLE 2:** Standardized redemption ratios by ATC class among segregated Roma colonies and complementary area.

ATC classification	Segregated roma colonies		Complementary area		Relative redemption (95%, CI)	Attributable number of redemptions	Attributable percentage of redemptions
	Dispensed/expected	SRR [95% CI]	Dispensed/expected	SRR [95% CI]			
A-alimentary tract and metabolism	5,107/4941.12	1.034 [1.006–1.062]	99,469/99634.87	0.998 [0.992–1.005]	1.035 (1.010–1.060)	173	3.5
B-blood and blood forming organs	1,894/1854.07	1.022 [0.977–1.069]	49,669/49708.93	0.999 [0.990–1.008]	1.022 (0.983–1.063)	43	−2.2
C-cardiovascular system	10,156/9136.16	1.112 [1.090–1.133]	274,969/275988.84	0.996 [0.993–1.001]	1.115 (1.097–1.135)	1,051	11.5
D-dermatologicals	685/710.4	0.964 [0.895–1.039]	8,575/8549.6	1.003 [0.982–1.024]	0.961 (0.904–1.022)	−28	−3.9
G-genito-urinary system and sex hormones	159/156.76	1.014 [0.868–1.185]	4,557/4559.22	0.999 [0.971–1.029]	1.015 (0.884–1.165)	2	−1.5
H-systemic hormonal preparations, excluding sex hormones and insulins	305/314.36	0.970 [0.867–1.085]	7,482/7472.64	1.001 [0.979–1.024]	0.969 (0.879–1.068)	−10	−3.1
J-anti-infectives for systemic use	3,716/4699.78	0.791 [0.766–0.817]	40,324/39340.2	1.025 [1.015–1.035]	0.771 (0.753–0.791)	−1,100	−22.9
M-musculo-skeletal system	3,936/3803.56	1.035 [1.003–1.067]	52,403/52535.44	0.997 [0.989–1.006]	1.037 (1.010–1.067)	141	3.7
N-nervous system	2,612/2573.89	1.015 [0.977–1.054]	49,299/49337.11	0.999 [0.990–1.008]	1.016 (0.982–1.051)	41	−1.6
P-antiparasitic products, insecticides and repellents	20/24.43	0.819 [0.528–1.269]	416/411.57	1.011 [0.918–1.113]	0.810 (0.573–1.145)	−5	−19.0
R-respiratory system	3,819/3736.66	1.022 [0.99–1.055]	45,222/45,304.34	0.998 [0.989–1.007]	1.024 (0.996–1.052)	90	−2.4
S-sensory organs	316/357.46	0.884 [0.792–0.987]	4,297/4255.54	1.010 [0.980–1.040]	0.875 (0.802–0.955)	−45	−12.5
V-various	995/928.99	1.071 [1.007–1.140]	4,268/4334.01	0.984 [0.955–1.014]	1.088 (1.028–1.151)	80	8.8
Total	33,720/32847.67	1.027 [1.016–1.038]	640,950/641822.33	0.999 [0.996–1.001]	1.028 (1.018–1.038)	920	2.8

SRR: standardized redemption ratio = observed/expected number of redemptions. Attributable number of redemptions = (SRR_segregated Roma colonies_–SRR_complementary area_) × expected number of redemptions in SRC.

Concerning the population-level impact, excess redemption was 1,051 prescriptions (11.5%) for cardiovascular agents, 173 (3.5%) for alimentary tract and metabolism-related drugs, and 141 (3.7%) for musculoskeletal system drugs in the SRC (Table 2). Inversely, for anti-infectives and sensory organ agents, 1,100 (22.9%) and 45 (12.5%) prescriptions were not redeemed in the SRC, respectively. In all, an excess of 920 (2.8%) redemptions was observed in the SRC compared to the CA.

## Discussion

### Main Findings in the International Context

This is the first study to investigate primary medication adherence among Roma people. We could evaluate redemption in the most vulnerable subgroup of Roma people who live in SRC by standardizing the redemption according to patient age and sex using Hungarian stratum-specific redemption ratios as a reference.

In general, the Hungarian crude redemption ratio (66.8%) was higher than those reported in the United States (60.7%) (Fernández et al., 2017) and Netherlands (48.5%) (van Geffen et al., 2009), but lower than the global estimate reported in a recent systematic review and meta-analysis (83%) (Cheen et al., 2019) and the crude redemption ratio reported in Portugal (77.2%) (da Costa et al., 2015) and Denmark (90.7%) (Pottegård et al., 2014). The discordance with a systematic review and meta-analysis might be due to firm follow up and good patient-physician relationship at comprehensive chronic care unit for chronic diseases. The positive effect of female sex on redemption in Hungary was found to be weak but statistically significant, which is consistent with observations from the United Kingdom, the United States and South Korea (Hagström et al., 2004; Farris et al., 2018; Lee et al., 2018). The relatively low redemption ratio among young and middle-aged Hungarian adults also fits the reported age dependency from other countries (Cooper et al., 1982; Mansur et al., 2009; Wenger et al., 2017).

Altogether, the observed primary medication adherence was better in the SRC than in the CA. The observed 2.8% excess corresponds to 920 redemptions a year in the population of 4,943 Roma people living in the SRC. Higher redemption rates in disadvantaged populations, as indicated by low levels of education and high level of deprivation, was observed previously in Hungarian studies (Boruzs et al., 2016; Boruzs et al., 2018; Harsha et al., 2019a; Harsha et al., 2019b; Juhász et al., 2020) and in other countries as well (Mauskop and Borden, 2011; Jackson et al., 2014; Rosicova et al., 2015; Ferdinand et al., 2017; Pandya and Bajorek, 2017; Lee et al., 2018; Gast and Mathes, 2019).

Cardiovascular drugs and alimentary tract and metabolism-related medications constituted the highest share of the prescriptions in both investigated populations. This reflects the prevalence of these chronic diseases in Hungary, as has been reported in previous studies (Balogh et al., 2010; Szigethy et al., 2012). Roma people living in the SRC exhibited better redemption of cardiovascular drugs (11.5%) and alimentary tract and metabolism-related drugs (3.5%) than those living in the CA, a difference that was confirmed by the standardized analysis.

Considering the number of prescriptions, the third most important ATC class is the group of anti-infectives for systemic use. The redemption inequality in this ATC group showed a reverse pattern: the redemption ratio was relatively high in the CA but lowest in the SRC, resulting in a significantly lower redemption ratio for the SRC. We found lower general redemption ratios in the SRC among children and the young adult age groups (Table 1), and a low relative importance of cardiovascular (0.7% of prescriptions, Supplementary Table 3C) and alimentary tract drugs (10.6% of prescriptions, Supplementary Table 3A) due to the low prevalence of chronic diseases among children and young adults. In addition, we observed a high importance of infections and anti-infective drugs among these groups (72.5% of prescriptions, Supplementary Table 3J). This lower redemption ratio among children could be the explanatory factor behind the observed deviation of the anti-infective redemption habit in the SRC from the general pattern. However, further investigation is needed to determine whether the causal mechanism behind this pattern is the impact of a Roma culture-related attitude toward children or the avoidance of antibiotic overuse among the Roma (Porco et al., 2012; Basu and Garg, 2018). The role of the poor living conditions determined high rate of infective disorders (reflected in the higher proportion of both prescription and redemption of antibiotics) in SRCs (Table 1) needs clarification as well.

### Practical Implications

Our study demonstrated that blaming the Roma for their poor health (beyond the fact that it is ethically unacceptable and it is a factor that contributes to the maintenance of their poor health) (European Union Agency for Fundamental Rights, 2009b; FRA/UNDP, 2013; Miranda et al., 2019) cannot be supported by data on primary medication adherence. Moreover, responsible health policy should consider the relatively good primary medication adherence of this population as an opportunity to organize culturally adapted interventions. When the poor healthcare utilization of this population is targeted by an intervention (Miranda et al., 2019), this factor should be added to the resources of the program. Our observations suggest that GPs should prefer medication based care instead of the non-medication based (life style modification, physiotherapy etc.) approaches for inhabitants of SRCs.

### Strengths and Limitations

This study used data from the NHIFM, which covers the whole country. Selection bias is likely very low, as we included all the residents’ prescriptions in the study area. Because, this database has no data on the use of dispensed medications, only the primary non-adherence could be evaluated. Further, our study due to the SRC vs CA comparison design could avoid all the uncertainties which are related to the person level identification of Roma participants.

However, our study has some limitations. Although our study recruited a large sample, this does not mean that it shows representative national Roma figures. The main limitation is that our results cannot be interpreted as an indicator of Roma vs non-Roma differences. On one hand, 6% of people living in SRC are non-Roma (Kósa et al., 2013). On the other hand, three-quarters of the Roma are living in the CA in Hungary (Teller, 2010). Consequently, our SRC-based analysis underestimated the real Roma-to-non-Roma differences.

A weakness of this study is that its dataset is from 2012. But the primary medication adherence of SRCs’ inhabitants had been not investigated before. It was not known whether the primary medication adherence is one of the risk factors for Roma people. The intervention program could build up the necessary database in 2012. Taking into consideration the significance of our results further studies are needed, obviously, to monitor the primary medication adherence among Roma people.

A prescription is proof that the medicine is needed for the patient. The confounding effect from the obvious influence of clinical status on the motivation of the patient to purchase the medicine is controlled by the prescription itself, as the prescription does fit the clinical status of the patient.

Although, price of drugs is one of the key factors that affect redemption, economic explanations for the observations could not be formulated in this investigation, because data on participants’ income were not collected to avoid the jeopardisation of trust between participants and implementation staff of the intervention program and data of the NHIF on co-payment for drug purchasing was not allowed to use in our study. Therefore, the complex issue of economic risk factors of primary medication adherence could not be investigated in our project. Their role needs further studies. It restricts the interpretation, but not the validity of our reported findings.

The availability of PHC in Hungary is among the best in Europe. Moreover, it has been reported that the Roma people living in SRC visit the GP more frequently than does the general population (Sándor et al., 2018). It appears improbable that limited access to primary care could somehow influence redemption practices.

## Conclusions

According to our observations, redemption of prescriptions was significantly higher (2.8%) among Roma people living in segregated colonies than among inhabitants of the same settlements’ complementary areas. The better redemption for cardiovascular (11.5%) and alimentary tract (3.5%) drugs was mainly responsible for this effect. It was not neutralized by the low redemption of anti-infective medicines (−23%) elicited by the critically low redemption among children and young adults.

These findings suggest that the primary medication adherence among Roma people living in segregations does not fit the general pattern reported by many studies that Roma people do not use health services properly and that prescription non-redemption is not responsible for the poor health of the Roma people. It also underlines the necessity of caution when cultural adaptations of interventions are planned and implemented for the Roma and that the in-depth study cannot be replaced by generalization of some distinct, even very convincing, findings. Moreover, our results suggest that the primary medication adherence of the Roma should be considered as an opportunity to develop their empowerment and to improve their health status.

## Data Availability

The data analyzed in this study is subject to the following licenses/restrictions: The drug consumption datasets of the National Health Insurance Fund are not available, in general. The research project had ocassional permission to use these data. The datasets generated and/or analyzed in our investigation are available from the corresponding author on reasonable request. Requests to access these datasets should be directed to janos.sandor@med.unideb.hu

## Abbreviations

ATC, anatomic therapeutic chemical; CA, complementary area; GP, general practitioner; NIHIFM, national institute of health insurance fund management; PHC, primary health care; RR, relative redemption; SRC, segregated roma colonies; SRR, standardized redemption ratio; WHO, world health organization
