# Supplementary material for: Is Prescription Nonredemption a Source of Poor Health Among the Roma? Cross-Sectional Analysis of Drug Consumption Data From the National Health Insurance Fund of Hungary
Source: Front Pharmacol. 2021 Mar 9;12:616092. doi: 10.3389/fphar.2021.616092 (PMC7985259; doi:10.3389/fphar.2021.616092)
Supplement: Supplementary file 1 [file datasheet1.docx]

***Supplementary Material***

**Supplementary table 1** National Crude Redemption ratios by sociodemographic characteristics in Hungary 2012

| **Patient characteristics** | | **Written prescriptions** | **Dispensed prescriptions** | **Dispensed percentage** | **p-value*** |
| --- | --- | --- | --- | --- | --- |
| Age groups (years) | 0-17 | 7,117,169 | 4,966,700 | 69.78 | <0.001 |
|  | 18-24 | 1,471,638 | 941,073 | 63.95 |  |
|  | 25-44 | 8,859,296 | 5,804,608 | 65.52 |  |
|  | 45-64 | 45,160,578 | 29,125,328 | 64.49 |  |
|  | 65 and above | 63,615,115 | 43,485,342 | 68.36 |  |
| Sex | Male | 50,581,743 | 33,589,698 | 66.41 | <0.001 |
|  | Female | 75,642,053 | 50,733,353 | 67.07 |  |
| Total | | 126,223,796 | 84,323,051 | 66.80 | - |

**by chi-square test*

**Supplementary table 2** National Crude Redemption ratios by ATC classifications in Hungary 2012

| **ATC groups** | **Written prescriptions** | **Dispensed prescriptions** | **Dispensed percentage** |
| --- | --- | --- | --- |
| A- Alimentary tract and metabolism | 20,395,783 | 14,417,501 | 70.69 |
| B- Blood and blood forming organs | 8,919,109 | 6,049,914 | 67.83 |
| C- Cardiovascular system | 61,978,491 | 39,118,918 | 63.12 |
| D- Dermatologicals | 1,519,730 | 958,432 | 63.07 |
| G- Genito-urinary system and sex hormones | 879,489 | 625,473 | 71.12 |
| H- Systemic hormonal preparations* | 1,306,624 | 929,123 | 71.11 |
| J- Anti-infectives for systemic use | 5,786,947 | 4,444,509 | 76.80 |
| M-Musculo-skeletal system | 7,849,077 | 5,544,645 | 70.64 |
| N- Nervous system | 7,944,060 | 5,730,169 | 72.13 |
| P-Antiparasitic products, insecticides and repellents | 62,044 | 46,736 | 75.33 |
| R-Respiratory system | 7,456,389 | 5,043,686 | 67.64 |
| S- Sensory organs | 871,650 | 597,112 | 68.50 |
| V-various | 1,254,403 | 816,833 | 65.12 |
| Total | 126,223,796 | 84,323,051 | 66.80 |

** excluding sex hormones and insulins*

**Supplementary table 3a** Crude prescription redemption ratio by sociodemographic characteristics for segregated Roma colonies and complementary area for ATC-A (Alimentary tract and metabolism group)

|  | | **Segregated Roma colonies** | | | **Complementary area** | | | **p-value *** |
| --- | --- | --- | --- | --- | --- | --- | --- | --- |
|  |  | Written prescriptions | Dispensed prescriptions | Dispensed percentage | Written prescriptions | Dispensed prescriptions | Dispensed percentage |  |
| Age category in years | 0-17 | 549 | 279 | 50.82 | 4,238 | 2,979 | 70.29 | <0.001 |
|  | 18-24 | 148 | 106 | 76.35 | 1,385 | 938 | 72.56 | 0.676 |
|  | 25-44 | 1,204 | 936 | 77.74 | 11,324 | 8,292 | 73.22 | 0.192 |
|  | 45-64 | 3,417 | 2,789 | 81.62 | 53,098 | 39,825 | 75.00 | 0.001 |
|  | 65 and above | 1,263 | 997 | 78.94 | 62,657 | 47,435 | 75.71 | 0.328 |
| Sex | Male | 2,496 | 1,944 | 77.88 | 48,788 | 36,512 | 74.84 | 0.198 |
|  | Female | 4,085 | 3,163 | 77.43 | 83,914 | 62,957 | 75.03 | 0.194 |
| Total | | 6,581 | 5,107 | 77.60 | 132,702 | 99,469 | 74.96 | 0.070 |

**by chi-square test*

**Supplementary table 3b** Crude prescription redemption ratio by sociodemographic characteristics for segregated Roma colonies and complementary area for ATC-B group (Blood and blood forming organs)

|  | | **Segregated Roma colonies** | | | **Complementary area** | | | **p-value *** |
| --- | --- | --- | --- | --- | --- | --- | --- | --- |
|  |  | Written prescriptions | Dispensed prescriptions | Dispensed percentage | Written prescriptions | Dispensed prescriptions | Dispensed percentage |  |
| Age category in years | 0-17 | 124 | 60 | 48.39 | 740 | 488 | 65.95 | 0.064 |
|  | 18-24 | 81 | 38 | 46.91 | 595 | 343 | 57.65 | 0.321 |
|  | 25-44 | 456 | 340 | 74.56 | 3,917 | 2,831 | 72.27 | 0.681 |
|  | 45-64 | 1,278 | 1,049 | 82.08 | 24,975 | 18,244 | 73.05 | 0.006 |
|  | 65 and above | 554 | 407 | 73.47 | 36,612 | 27,763 | 75.83 | 0.630 |
| Sex | Male | 954 | 754 | 79.04 | 27,793 | 20,635 | 74.25 | 0. 207 |
|  | Female | 1,539 | 1,140 | 74.07 | 39,046 | 29,034 | 74.36 | 0.923 |
| Total | | 2,493 | 1,894 | 75.97 | 66,839 | 49,669 | 74.31 | 0.476 |

**by chi-square test*

**Supplementary table 3c** Crude prescription redemption ratio by sociodemographic characteristics for segregated Roma colonies and complementary area for ATC-C group (Cardiovascular system)

|  | | **Segregated Roma colonies** | | | **Complementary area** | | | **p-value *** |
| --- | --- | --- | --- | --- | --- | --- | --- | --- |
|  |  | Written prescriptions | Dispensed prescriptions | Dispensed percentage | Written prescriptions | Dispensed prescriptions | Dispensed percentage |  |
| Age category in years | 0-17 | 28 | 21 | 75.00 | 150 | 110 | 73.33 | 0.943 |
|  | 18-24 | 69 | 43 | 62.32 | 1,001 | 720 | 71.93 | 0.474 |
|  | 25-44 | 2,235 | 1,687 | 75.48 | 25,065 | 16,477 | 65.74 | <0.001 |
|  | 45-64 | 7,350 | 5,625 | 76.53 | 170,792 | 111,962 | 65.55 | <0.001 |
|  | 65 and above | 3,874 | 2,780 | 71.76 | 212,497 | 145,700 | 68.57 | 0.069 |
| Sex | Male | 4,852 | 3,581 | 73.80 | 155,851 | 103,520 | 66.42 | <0.001 |
|  | Female | 8,704 | 6,575 | 75.54 | 253,654 | 171,449 | 67.59 | <0.001 |
| Total | | 13,556 | 10,156 | 74.92 | 409,505 | 274,969 | 67.15 | <0.001 |

**by chi-square test*

**Supplementary table 3d** Crude prescription redemption ratio by sociodemographic characteristics for segregated Roma colonies and complementary area for ATC-D group (Dermatologicals)

|  | | **Segregated Roma colonies** | | | **Complementary area** | | | **p-value *** |
| --- | --- | --- | --- | --- | --- | --- | --- | --- |
|  |  | Written prescriptions | Dispensed prescriptions | Dispensed percentage | Written prescriptions | Dispensed prescriptions | Dispensed percentage |  |
| Age category in years | 0-17 | 276 | 156 | 56.52 | 1,975 | 1,241 | 62.84 | 0.320 |
|  | 18-24 | 49 | 33 | 67.35 | 716 | 509 | 71.09 | 0.816 |
|  | 25-44 | 238 | 145 | 60.92 | 2,221 | 1,551 | 69.83 | 0.216 |
|  | 45-64 | 312 | 239 | 76.60 | 4,239 | 2,987 | 70.46 | 0.349 |
|  | 65 and above | 154 | 112 | 72.73 | 3,233 | 2,287 | 70.74 | 0.827 |
| Sex | Male | 442 | 261 | 59.05 | 4,784 | 3,260 | 68.14 | 0.078 |
|  | Female | 587 | 424 | 72.23 | 7,600 | 5,315 | 69.93 | 0.625 |
| Total | | 1,029 | 685 | 66.57 | 12,384 | 8,575 | 69.24 | 0.443 |

**by chi-square test*

**Supplementary table 3g** Crude prescription redemption ratio by sociodemographic characteristics for segregated Roma colonies and complementary area for ATC-G group (Genito-urinary system and sex hormones)

|  | | **Segregated Roma colonies** | | | **Complementary area** | | | **p-value *** |
| --- | --- | --- | --- | --- | --- | --- | --- | --- |
|  |  | Written prescriptions | Dispensed prescriptions | Dispensed percentage | Written prescriptions | Dispensed prescriptions | Dispensed percentage |  |
| Age category in years | 0-17 | 5 | 4 | 80.00 | 39 | 33 | 84.62 | 0.937 |
|  | 18-24 | 10 | 5 | 50.00 | 120 | 91 | 75.83 | 0.458 |
|  | 25-44 | 43 | 34 | 79.07 | 455 | 380 | 83.52 | 0.819 |
|  | 45-64 | 77 | 55 | 71.43 | 1,573 | 1,162 | 73.87 | 0.852 |
|  | 65 and above | 66 | 61 | 92.42 | 3,659 | 2,891 | 79.01 | 0.381 |
| Sex | Male | 96 | 81 | 84.38 | 3,400 | 2,754 | 81.00 | 0.790 |
|  | Female | 105 | 78 | 74.29 | 2,446 | 1,803 | 73.71 | 0.960 |
| Total | | 201 | 159 | 79.10 | 5,846 | 4,557 | 77.95 | 0.892 |

**by chi-square test*

**Supplementary table 3h** Crude prescription redemption ratio by sociodemographic characteristics for segregated Roma colonies and complementary area for ATC-H group (Systemic hormonal preparations**)

|  | | **Segregated Roma colonies** | | | **Complementary area** | | | **p-value *** |
| --- | --- | --- | --- | --- | --- | --- | --- | --- |
|  |  | Written prescriptions | Dispensed prescriptions | Dispensed percentage | Written prescriptions | Dispensed prescriptions | Dispensed percentage |  |
| Age category in years | 0-17 | 11 | 9 | 81.82 | 149 | 115 | 77.18 | 0.900 |
|  | 18-24 | 7 | 3 | 42.86 | 189 | 158 | 83.60 | 0.330 |
|  | 25-44 | 105 | 76 | 72.38 | 1,458 | 1,135 | 77.85 | 0.640 |
|  | 45-64 | 221 | 163 | 73.76 | 4,364 | 3,348 | 76.72 | 0.710 |
|  | 65 and above | 62 | 54 | 87.10 | 3,491 | 2,726 | 78.09 | 0.561 |
| Sex | Male | 96 | 70 | 72.92 | 1,860 | 1,428 | 76.77 | 0.749 |
|  | Female | 310 | 235 | 75.81 | 7,791 | 6,054 | 77.71 | 0.779 |
| Total | | 406 | 305 | 75.12 | 9,651 | 7,482 | 77.53 | 0.684 |

**by chi-square test*

**** *excluding sex hormones and insulins*

**Supplementary table 3j** Crude prescription redemption ratio by sociodemographic characteristics for segregated Roma colonies and complementary area for ATC-J group (Anti-infectives for systemic use)

|  | | **Segregated Roma colonies** | | | **Complementary area** | | | **p-value *** |
| --- | --- | --- | --- | --- | --- | --- | --- | --- |
|  |  | Written prescriptions | Dispensed prescriptions | Dispensed percentage | Written prescriptions | Dispensed prescriptions | Dispensed percentage |  |
| Age category in years | 0-17 | 4,186 | 2,105 | 50.29 | 20,819 | 13,736 | 65.98 | <0.001 |
|  | 18-24 | 490 | 182 | 37.14 | 5,195 | 3,126 | 60.17 | <0.001 |
|  | 25-44 | 731 | 531 | 72.64 | 10,005 | 7,883 | 78.79 | 0.168 |
|  | 45-64 | 851 | 730 | 85.78 | 11,760 | 10,134 | 86.17 | 0.930 |
|  | 65 and above | 193 | 168 | 87.05 | 6,220 | 5,445 | 87.54 | 0.958 |
| Sex | Male | 2,807 | 1,562 | 55.65 | 22,574 | 16,439 | 72.82 | <0.001 |
|  | Female | 3,644 | 2,154 | 59.11 | 31,425 | 23,885 | 76.01 | <0.001 |
| Total | | 6,451 | 3,716 | 57.60 | 53,999 | 40,324 | 74.68 | <0.001 |

**by chi-square test*

**Supplementary table 3m** Crude prescription redemption ratio by sociodemographic characteristics for segregated Roma colonies and complementary area for ATC-M group (Musculo-skeletal system)

|  | | **Segregated Roma colonies** | | | **Complementary area** | | | **p-value *** |
| --- | --- | --- | --- | --- | --- | --- | --- | --- |
|  |  | Written prescriptions | Dispensed prescriptions | Dispensed percentage | Written prescriptions | Dispensed prescriptions | Dispensed percentage |  |
| Age category in years | 0-17 | 491 | 297 | 60.49 | 2,773 | 1,819 | 65.60 | 0.308 |
|  | 18-24 | 136 | 74 | 54.41 | 1,089 | 666 | 61.16 | 0.444 |
|  | 25-44 | 1,228 | 914 | 74.43 | 9,571 | 6,867 | 71.75 | 0.430 |
|  | 45-64 | 2,532 | 2,076 | 81.99 | 31,593 | 23,191 | 73.41 | <0.001 |
|  | 65 and above | 756 | 575 | 76.06 | 26,010 | 19,860 | 76.36 | 0.945 |
| Sex | Male | 1,853 | 1,354 | 73.07 | 28,110 | 20,638 | 73.42 | 0.898 |
|  | Female | 3,290 | 2,582 | 78.48 | 42,926 | 31,765 | 74.00 | 0.031 |
| Total | | 5,143 | 3,936 | 76.53 | 71,036 | 52,403 | 73.77 | 0.094 |

**by chi-square test*

**Supplementary table 3n** Crude prescription redemption ratio by sociodemographic characteristics for segregated Roma colonies and complementary area for ATC-N group (Nervous system)

|  | | **Segregated Roma colonies** | | | **Complementary area** | | | **p-value *** |
| --- | --- | --- | --- | --- | --- | --- | --- | --- |
|  |  | Written prescriptions | Dispensed prescriptions | Dispensed percentage | Written prescriptions | Dispensed prescriptions | Dispensed percentage |  |
| Age category in years | 0-17 | 284 | 224 | 78.87 | 929 | 762 | 82.02 | 0.701 |
|  | 18-24 | 103 | 69 | 66.99 | 638 | 459 | 71.94 | 0.670 |
|  | 25-44 | 869 | 665 | 76.52 | 6,911 | 5,653 | 81.80 | 0.222 |
|  | 45-64 | 1,588 | 1,275 | 80.29 | 23,352 | 17,427 | 74.63 | 0.060 |
|  | 65 and above | 525 | 379 | 72.19 | 32,748 | 24,998 | 76.33 | 0.411 |
| Sex | Male | 1,161 | 921 | 79.33 | 20,796 | 16,346 | 78.60 | 0.839 |
|  | Female | 2,208 | 1,691 | 76.59 | 43,782 | 32,953 | 75.27 | 0.600 |
| Total | | 3,369 | 2,612 | 77.53% | 64,578 | 49,299 | 76.34 | 0.563 |

**by chi-square test*

**Supplementary table 3p** Crude prescription redemption ratio by sociodemographic characteristics for segregated Roma colonies and complementary area for ATC-P group (Antiparasitic products, insecticides and repellents)

|  | | **Segregated Roma colonies** | | | **Complementary area** | | | **p-value *** |
| --- | --- | --- | --- | --- | --- | --- | --- | --- |
|  |  | Written prescriptions | Dispensed prescriptions | Dispensed percentage | Written prescriptions | Dispensed prescriptions | Dispensed percentage |  |
| Age category in years | 0-17 | 4 | 3 | 75.00 | 24 | 23 | 95.83 | 0.764 |
|  | 18-24 | 8 | 5 | 62.50 | 28 | 17 | 60.71 | 0.964 |
|  | 25-44 | 7 | 5 | 71.43 | 151 | 105 | 69.54 | 0.964 |
|  | 45-64 | 10 | 5 | 50.00 | 245 | 202 | 82.45 | 0.364 |
|  | 65 and above | 3 | 2 | 66.67 | 91 | 69 | 75.82 | 0.889 |
| Sex | Male | 16 | 10 | 62.50 | 179 | 119 | 66.48 | 0.883 |
|  | Female | 16 | 10 | 62.50 | 360 | 297 | 82.50 | 0.498 |
| Total | | 32 | 20 | 62.50 | 539 | 416 | 77.18 | 0.470 |

**by chi-square test*

**Supplementary table 3r** Crude prescription redemption ratio by sociodemographic characteristics for segregated Roma colonies and complementary area for ATC-R group (Respiratory system)

|  | | **Segregated Roma colonies** | | | **Complementary area** | | | **p-value *** |
| --- | --- | --- | --- | --- | --- | --- | --- | --- |
|  |  | Written prescriptions | Dispensed prescriptions | Dispensed percentage | Written prescriptions | Dispensed prescriptions | Dispensed percentage |  |
| Age category in years | 0-17 | 1,505 | 913 | 60.66 | 9,886 | 6,718 | 67.95 | 0.011 |
|  | 18-24 | 165 | 107 | 64.85 | 2,822 | 1,857 | 65.80 | 0.909 |
|  | 25-44 | 683 | 541 | 79.21 | 8,106 | 5,582 | 68.86 | 0.020 |
|  | 45-64 | 2,135 | 1,747 | 81.83 | 22,677 | 16,551 | 72.99 | 0.001 |
|  | 65 and above | 637 | 511 | 80.22 | 18,646 | 14,514 | 77.84 | 0.618 |
| Sex | Male | 2,073 | 1,513 | 72.99 | 26,910 | 19,835 | 73.71 | 0.779 |
|  | Female | 3,052 | 2,306 | 75.56 | 35,227 | 25,387 | 72.07 | 0.100 |
| Total | | 5,125 | 3,819 | 74.52 | 62,137 | 45,222 | 72.78 | 0.289 |

**by chi-square test*

**Supplementary table 3s** Crude prescription redemption ratio by sociodemographic characteristics for segregated Roma colonies and complementary area for ATC-S group (Sensory organs)

|  | | **Segregated Roma colonies** | | | **Complementary area** | | | **p-value *** |
| --- | --- | --- | --- | --- | --- | --- | --- | --- |
|  |  | Written prescriptions | Dispensed prescriptions | Dispensed percentage | Written prescriptions | Dispensed prescriptions | Dispensed percentage |  |
| Age category in years | 0-17 | 205 | 97 | 47.32 | 1,125 | 748 | 66.49 | 0.001 |
|  | 18-24 | 18 | 6 | 33.33 | 276 | 179 | 64.86 | 0.160 |
|  | 25-44 | 82 | 59 | 71.95 | 878 | 622 | 70.84 | 0.931 |
|  | 45-64 | 121 | 96 | 79.34 | 1,773 | 1,271 | 71.69 | 0.473 |
|  | 65 and above | 79 | 58 | 73.42 | 1,960 | 1,477 | 75.36 | 0.882 |
| Sex | Male | 204 | 129 | 63.24 | 2,248 | 1,634 | 72.69 | 0.234 |
|  | Female | 301 | 187 | 62.13 | 3,764 | 2,663 | 70.75 | 0.178 |
| Total | | 505 | 316 | 62.57 | 6,012 | 4,297 | 71.47 | 0.074 |

**by chi-square test*

**Supplementary table 3v** Crude prescription redemption ratio by sociodemographic characteristics for segregated Roma colonies and complementary area for ATC-V group (Various)

|  | | **Segregated Roma colonies** | | | **Complementary area** | | | **p-value *** |
| --- | --- | --- | --- | --- | --- | --- | --- | --- |
|  |  | Written prescriptions | Dispensed prescriptions | Dispensed percentage | Written prescriptions | Dispensed prescriptions | Dispensed percentage |  |
| Age category in years | 0-17 | 1,171 | 966 | 82.49 | 4,325 | 3,415 | 78.96 | 0.373 |
|  | 18-24 | 0 | 0 | - | 76 | 59 | 77.63 | NA |
|  | 25-44 | 3 | 0 | 0 | 146 | 75 | 51.37 | 0.216 |
|  | 45-64 | 31 | 25 | 80.65 | 395 | 277 | 70.13 | 0.617 |
|  | 65 and above | 11 | 4 | 36.36 | 731 | 442 | 60.47 | 0.381 |
| Sex | Male | 612 | 523 | 85.46 | 2,796 | 2,159 | 77.22 | 0.125 |
|  | Female | 604 | 472 | 78.15 | 2,877 | 2,109 | 73.31 | 0.346 |
| Total | | 1,216 | 995 | 81.83 | 5,673 | 4,268 | 75.23 | 0.076 |

**by chi-square test, NA- Not applicable*
